# Editorial: Women in NK and innate lymphoid cell biology

**DOI:** 10.3389/fimmu.2023.1157166

**Published:** 2023-02-15

**Authors:** Alessandra Zingoni, Teresa Bellón

**Affiliations:** ^1^ Laboratory affiliated to Istituto Pasteur Italia-Fondazione Cenci Bolognetti, Department of Molecular Medicine, “Sapienza” University of Rome, Rome, Italy; ^2^ Drug Hypersensitivity and Innate Immunology Laboratory, University Hospital La Paz Health Research Institute (IdiPaz), Madrid, Spain

**Keywords:** NK cell, NK cell development, uterine (u)NK cells, NK cells and cancer, NK cells and Sars-Cov-infection

At present, less than 30% of researchers worldwide are women. Long-standing biases and gender stereotypes are discouraging girls and women away from science-related fields. Science and gender equality are, however, essential to ensure sustainable development as highlighted by UNESCO. In order to change traditional mindsets, gender equality must be promoted, stereotypes defeated, and girls and women should be encouraged to pursue careers as researcher. Therefore, Frontiers in Immunology is proud to offer this platform to promote the work of women scientists, across all fields of NK and Innate Lymphoid Cell Biology. The work presented here highlights the diversity of research performed across the entire breadth of NK and Innate Lymphoid Cell Biology research and presents advances in theory, experiment, and methodology with applications to compelling problems. Together, these 8 articles having women as first or last authors deal with distinct aspects of NK cell biology in health and disease.

## NK cell development

Three articles address different aspects of NK cell development.

In the manuscript by the research group directed from Wu et al., autologous transplantation of rhesus macaque hematopoietic stem cells (HSCs) was performed to track the clonal distribution of T, B, myeloid and NK cell populations in blood and across tissues, including liver, spleen, lung, and gastrointestinal (GI) tract. The experimental setting relies on clonal tracking *via* introduction of high diversity genetic barcodes together with GFP into HSCs to follow immune cell development. Interestingly, authors observed tissue resident (TR) NK cell clones specific for some tissues such as lung or GI tract as well as TR NK cell clones shared across liver and spleen, and distinct from other tissues. This experimental model could be further utilized to study clonal changes in circulating NK cells and in NK cells infiltrating tissues in response to infections, vaccines, or immunotherapy.

Persyn et al. studied the role of the transcription factor interferon regulatory factor (IRF)-2 in human NK cell differentiation. As such, *in vitro* NK cell differentiation cultures were settled by starting from cord blood-derived hematopoietic stem cells (HSC) transduced with IRF2 knockdown or IRF2 overexpression vectors. Of interest, authors found that IRF2 knockdown strongly reduced NK cell proliferation during development. Also, they demonstrated that IRF2 is necessary for functional maturation of NK cells, as NK IRF2^-/-^cells had a less mature phenotype and showed decreased cytotoxic potential, as well as a greatly reduced cytokine secretion. Thus, IRF2 plays an important role during development and functional maturation of human NK cells.

Another cutting edge topic that was addressed by Bourayou and Golub aims at explaining how inflammation could impact on NK cell development, maturation and effector functions. In particular, the authors firstly summarized known and emerging factors implicated in the regulation of NK cell maturation and then discussed how distinct inflammatory conditions could influence this process.

## Uterine NK cells

NK cells have emerged as crucial innate immune cells involved in the physiology and pathology of both non-pregnant and pregnant uteri. Xie et al. have elaborated an interesting overview about past and recent advances in human uterine NK (uNK) cell research highlighting the role of uNK cells in both normal physiological conditions and in pregnancy. The mechanism of action of uNK cells in various reproductive diseases including recurrent miscarriage, preeclampsia, and endometriosis is discussed from multiple perspectives. In addition, the potential clinical usage of uNK cells as a novel immunotherapeutic strategy for reproductive disorders is largely described.

## NK cells and SARS-CoV-infection

In the manuscript by Fionda et al., the potential impact of age-related changes on NK cell phenotype and function during SARS-CoV-2 infection was investigated. Of interest, adult patients (below 65 years of age) had a reduced number of total NK cells, while elderly (over 65 years of age) showed a peculiar skewing of NK cell subsets towards the CD56^low^CD16^high^ and CD56^neg^ phenotypes, expressing activation markers and check-point inhibitory receptors. IFN-γ production was severely compromised only in adult patients in a TGF-β−dependent manner. Authors suggest that TGF-β might limit an excessive NK cell activation that could amplify the systemic inflammatory response leading to a less severe infection course.

## NK cells in head and neck carcinoma

In a model of head and neck squamous cell carcinoma (HNSCC), tumor infiltrating NK cells were defined based on the analysis of cell surface markers and on functional assays (Mele et al.). The results obtained showed a NK cell phenotype predominantly characterized by increased expression of the inhibitory receptor NKG2A and by a concomitant reduction of a number of activating receptors (i.e: NKG2D, NKp30 and CD16). This *inhibitory* phenotype was associated with a reduced antibody-dependent cellular cytotoxicity (ADCC) activity evaluated either as degranulation or as IFN-γ production. An interesting observation derived from this work concerns an increment of glucocorticoid-induced tumor necrosis factor-related (GITR) costimulatory receptor not only on NK cells but also on tumor infiltrating CD4 and CD8 T lymphocytes. Thus, GITR could represent a potential therapeutic target for immunotherapeutic approaches combining GITR agonists and immune checkpoint inhibitors.

## NK cell functional activity and targeted therapies

Mestre-Duran et al. showed that the functional capabilities and phenotype of NK cells activated *in vitro* through TLR4/9 agonists were not completely abolished by the inhibition of the JAK-STAT pathway by ruxolitinib. This drug has been recently approved for treating acute and chronic graft-versus-host disease. These findings could have clinical implications for the protective role of NK cells in the *graft versus leukemia* effect after bone marrow allogenic transplantation.

## NKT cells in adenomyosis

In the manuscript by Chen et al., a novel cluster of cyotoxic lymphocytes sharing T and NK cell features and expressiong high levels of secreted frizzled related protein 4 (SFRP4) and insulin-like growth factor binding protein 5 (IGFBP5) (i.e: SFRP4^+^IGFBP5^hi^NKT cells) was discovered by single cell RNAseq analysis in the foci of patients with adenomyosis, an estrogen-dependent gynecological disease, in which chronic pain is a clinically unresolved issue.

Of interest, it was observed that these SFRP4^+^IGFBP5^hi^NKT cells were capable of converting part of the stem cells found in affected tissues into neurogenic cells and were therefore, likely responsible of inducing pain.

## Author contributions

All authors listed have made a substantial, direct, and intellectual contribution to the work and approved it for publication.

